# Seroepidemiological Study of Spotted Fever Group Rickettsiae and Identification of a Putative New Species, *Rickesttsia* sp. Da-1, in Gongliao, Northeast Taiwan

**DOI:** 10.3390/pathogens10111434

**Published:** 2021-11-04

**Authors:** Tsai-Ying Yen, Hsi-Chieh Wang, Yin-Chao Chang, Chien-Ling Su, Shu-Fen Chang, Pei-Yun Shu, Kun-Hsien Tsai

**Affiliations:** 1 Institute of Environmental and Occupational Health Sciences, College of Public Health, National Taiwan University, Taipei 100025, Taiwan; d00849002@ntu.edu.tw (T.-Y.Y.); sjwang1019@gmail.com (H.-C.W.); 2 Dr. Enjoy’s Clinic, Gong-Liao District, New Taipei City 228003, Taiwan; chapter767474@gmail.com; 3 Center for Diagnostics and Vaccine Development, Centers for Disease Control, Ministry of Health and Welfare, Taipei 115210, Taiwan; sue@cdc.gov.tw (C.-L.S.); vivi@cdc.gov.tw (S.-F.C.); 4 Department of Public Health, College of Public Health, National Taiwan University, Taipei 100025, Taiwan

**Keywords:** spotted fever group rickettsiae, seroprevalence, *Dermacentor auratus*, *Rickettsia* sp. Da-1

## Abstract

Tick-borne spotted fever group (SFG) rickettsioses were neglected in Taiwan. The study reported a seroepidemiological survey of SFG rickettsiae in residents in Gongliao District, Northeast Taiwan. Blood samples were examined for antibodies against SFG rickettsiae by enzyme-linked immunosorbent assay and immunofluorescence assay. Risk factors were assessed using logistic regression. Ticks parasitizing dogs were collected within a 2 km radius from the houses of seropositive participants, and PCR was performed to detect possible tick-borne pathogens. Of 1108 participants, 75 (6.8%) had antibodies against SFG rickettsiae. Residents were more likely to be seropositive if they were older than 65 years, recruited by Dr. Enjoy’s Clinic, or resided in Jilin village. A total of 184 ticks including 5 species (*Rhipicephalus sanguineus*, *Rhipicephalus haemaphysaloides*, *Dermacentor auratus*, *Haemaphysalis hystricis*, *Haemaphysalis ornithophila*) were collected. *Rickettsia* spp. were detected in 6.5% (12/184) of ticks. *Rickettsia* sp. TwKM01 was found in 6 *R. sanguineus* and 4 *R. haemaphysaloides*; while *Rickettsia* sp. TwKM03 was identified in 1 *R. sanguineus*. Moreover, gene-based pairwise analysis indicated identification of a putative new species, *Rickettsia* sp. Da-1, in *D. auratus*. These findings provided evidence of SFG rickettsiae infection in ticks and suggested SFG rickettsiae exposure in the residents.

## 1. Introduction

Rickettsiae are obligate intracellular Gram-negative bacteria belonging to the order Rickettsiales [1]. Genus *Rickettsia* was classified into 3 groups: spotted fever group (SFG) rickettsiae, typhus group (TG) rickettsiae, and scrub typhus group based on serology [2]. Later, the scrub typhus group was designated as a new genus, *Orientia* [3]. Further genetic evidence has suggested the addition of a transitional group (TRG) along with the ancestral group (AG) rickettsiae [4]. SFG rickettsiae and AG rickettsiae are primarily transmitted by ticks; while TG rickettsiae and TRG rickettsiae are associated with fleas, chiggers, or lice [5].

In Taiwan, the notifiable rickettsial diseases cover scrub typhus, epidemic typhus, and murine typhus. Scrub typhus and murine typhus led to 300–500 and 20–60 confirmed cases each year, respectively; while epidemic typhus has not been documented since World War II [6]. The fact that only 13.1–19.9% of the blood samples sent to the Taiwan CDC for laboratory diagnosis of scrub typhus were actually positive for *Orientia tsutsugamushi* infection suggests other microorganisms were involved in causing the illness [6]. Indeed, human cases of granulocytic anaplasmosis (11/274, 4.0%) has been demonstrated from these samples, and cases of human monocytic ehrlichiosis were recently identified [7,8,9]. Infection of *Rickettsia felis*, a *Rickettsia* now belonging to the TRG, has also been reported in 0.7–3.3% of patients with fever of unknown origin [10,11,12]. SFG rickettsiae infection shares similar clinical features with scrub typhus [1]; however, only one record of imported *Rickettsia africae* infection has been described in Taiwan [13]. Contradictorily, SFG rickettsiae were common in the field. *Rickettsia conorii* has been found in 7.1–50.0% of *Ixodes granulatus*, 0.9% (1/109) of *Rhipicephalus haemaphysaloides*, and 1.3% (1/80) of *Stilvalius aporus* fleas in Central and East Taiwan and on the off-shore islands [14,15]. *Rickettsia japonica* was detected in *Haemaphysalis bandicoda* (7/10, 70.0%) and *S. aporus* (2/80, 2.5%) in South and East Taiwan, as well as *Rickettsia rickettsii* in *I. granulatus* (1.1%–9.4%) and *R. haemaphysaloides* (4/109, 3.7%) on the offshore islands [14,15]. *Rickettsia helvetica* and *Rickettsia monacensis* were identified in *Ixodes columnae* (8/17, 47.1%) and *Ixodes nipponensis* (1/1, 100%) collected from birds [16]. *Rickettsia parkeri*-like species were found in *I. granulatus* in Kinmen (12/247, 4.9%) [17]. Infection of small mammals by *R. conorii*, *R. japonica*, *R. rickettsii*, and *Rickettsia raoultii* were demonstrated with serological or molecular evidence [14,18,19]. Besides, a growing number of new species, including *Rickettsia* sp. TwKM01, *Rickettsia* sp. IG-1, *Rickettsia* sp. RR01, *Rickettsia* sp. TwKM02, and *Rickettsia* sp. TwKM03, have been identified in *R. haemaphysaloides*, *S. aporus*, *I. granulatus*, *Rhipicephalus sanguineus*, and *Leptotrombidium deliense* chigger mites, in Central and East Taiwan and on the offshore islands [20,21,22]. The diverse and widespread of *Rickettsia* spp. should serve as a warning sign to human infection of rickettsial diseases. Thus, we have reason to believe that SFG rickettsiae infection is unrecognized and underestimated in Taiwan.

Ticks are important medically not only because they are ectoparasites of vertebrates, but they can transmit various pathogens [23]. Approximately 896 species of ticks belonging to 3 families, Argasidae, Ixodidae, and Nuttalliellidae, were recognized worldwide [24]. Thirty-nine tick species occur in Taiwan [16,25,26,27]. Parasitic genera such as *Anaplasma*, *Babesia*, *Borrelia*, *Ehrlichia*, and *Rickettsia* have been detected in *R. haemaphysaloides*, *Haemaphysalis* *ornithophila*, *R. sanguineus*, *I. granulatus*, *Ixodes* *ovatus*, *Ixodes* *turdus*, *Haemaphysali* *flava*, *I. columnae*, and *I. nipponensis*, suggesting the risk of infection by most of the identified tick-borne pathogens in people in Taiwan [16,28,29,30]. However, tick-borne diseases were reported scarcely [7,8,9,31,32]. In fact, most of the surveys were conducted on off-shore islands, and information about regional abundance of tick species was limited. Moreover, recent discovery of severe fever with thrombocytopenia syndrome virus in *Rhipicephalus microplus* as well as the first human case highlighted the importance of continuous surveillance of ticks and tick-borne diseases [33,34].

An early survey conducted in Tainan detected prevalence of 3.5–4.4% for antibodies against SFG rickettsiae, and another research also conducted in the southern Taiwan showed a seropositive rate of 2.9% (12/413) for SFG rickettsiae antibodies in patients suspected with Q fever, scrub typhus, murine typhus, leptospirosis, and dengue fever [11,35]. In order to clarify the extent of human SFG rickettsiae infection, here we report a cross-sectional study of the seroprevalence of SFG rickettsiae in residents of Gongliao District in New Taipei City, Taiwan. Potential tick vectors were collected, and infection of pathogens were screened to evaluate the risks of tick-borne diseases in the region.

## 2. Results

### 2.1. Demographics of the Participants

A total of 1108 blood samples were collected during January to December in 2008. The participants were aged 12 to 104 (median age 64 years), and the average age of subjects from Dr. Enjoy’s Clinic was slightly older than those from the other two sources (*p* < 0.01). More than half of the participants were females (571/1108, 51.5%). Most samples from males were collected from patients visiting the group practice center (*p* < 0.01). The study population resided in 11 villages of Gongliao District. However, majority of the subjects enrolled by the group practice center inhabited the northeastern Gongliao and the coastal villages; while subjects enrolled by Dr. Enjoy’s Clinic mostly lived in the southwestern Gongliao (Table 1).

### 2.2. Serology

**Figure 1:**
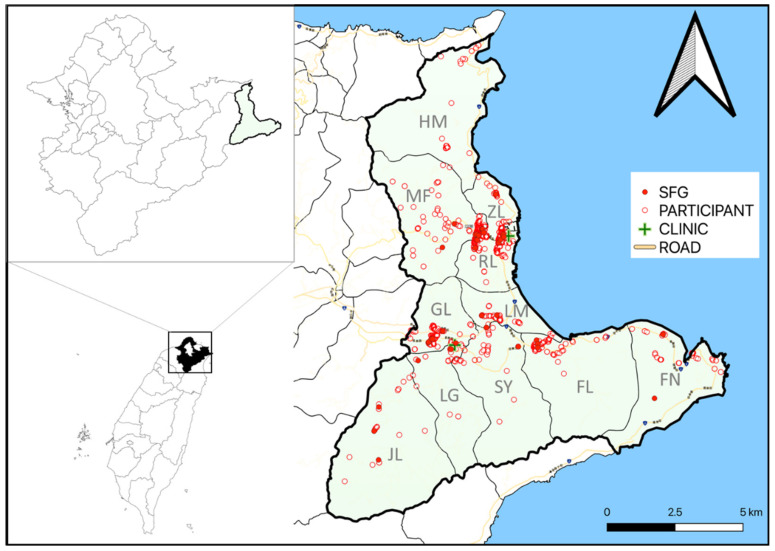
Location of samples positive for IgG antibodies against spotted fever group rickettsiae. GL: Gongliao Village; JL: Jilin Village; SY: Shuangyu Village; LG: Longgang Village; LM: Longmen Village; FL: Fulong Village; RL: Renli Village; ZL: Zhenli Village; FN: Fulian Village; MF: Meifeng Village; HM: Hemei Village.

Of 1108 serum samples, 118 (118/1108, 10.6%) were positive for SFG rickettsiae antibodies by enzyme-linked immunosorbent assay (ELISA). IgG against *R. rickettsii* was detected in 77 (77/1108, 6.9%) samples by immunofluorescence assay (IFA), and 75 of them were positive by both IFA and SFGR ELISA, resulting in a seropositive rate of 6.8% (75/1108) for SFG rickettsiae. Besides, 68 (68/1108, 6.1%) samples had antibodies reacting to *R. conorii* (Table 2). The highest prevalence of IgG against SFG rickettsiae was identified in the participants from Jilin village (Figure 1). 

Given that scrub typhus is the most reported rickettsial disease in Taiwan and cross-reactivity occurs between rickettsiae, antibodies to *R. typhi* and *O. tsutsugamushi* were examined for further comparison. Antibodies against *Rickettsia typhi* were found in 45 (45/1108, 4.1%) samples; while 155 (155/1108, 14.0%) samples were seropositive to *O. tsutsugamushi*. Forty-three sera reacted to more than one group of rickettsiae. Of them, 23 (23/1108, 2.1%) had antibodies against SFG rickettsiae (*R. rickettsii*) and TG rickettsiae; 5 (5/1108, 0.5%) had antibodies against SFG rickettsiae (*R. rickettsii*) and *O. tsutsugamushi*; 5 (5/1108, 0.5%) had antibodies against TG rickettsiae and *O. tsutsugamushi*; 10 (10/1108), 0.9%) reacted to SFG rickettsiae (*R. rickettsii*), TG rickettsiae, and *O. tsutsugamushi*.

### 2.3. Potential Risk Factors for SFG Rickettsiae Exposure

Univariable logistic regression revealed significantly positive association between SFG rickettsiae IgG seropositivity and older age, patients visiting either the group practice center or Dr. Enjoy’s Clinic versus healthy individuals, living in Jilin village, and the job of industrial laborer (Table 3). In multivariable logistic regression, seropositivity remained significantly associated with age (*p* = 0.014). Sera positive for IgG against SFG rickettsiae were 2.1 times more likely to be collected from participants ≥ 65 years-old (95% CI = 1.2–3.8) and 3.3 times more likely to be from inhabitants of Jilin village. Reactivity to multiple groups of rickettsiae was also positively associated with older age (*p* = 0.009) and patients of the group practice center (95% CI = 1.2–53.3) or Dr. Enjoy’s Clinic (95% CI = 2.8–255.6).

### 2.4. Collection of Ticks

To further understand the transmission of SFG rickettsiae in the area, ticks were collected from dogs living within 2 kilometer radius from the participants having IgG against SFG rickettsiae. Of 72 dogs encountered, ticks were found on 14 dogs, giving an infestation rate of 19.4% (14/72). A total of 184 ticks were collected including 155 *R. sanguineus*, 24 *R. haemaphysaloides*, 1 *Dermacentor auratus* (MZ823781), 3 *H. hystricis* (MZ823778), and 1 *H. ornithophila* (MZ823776). 

### 2.5. Molecular Findings in Ticks

**Figure 2:**
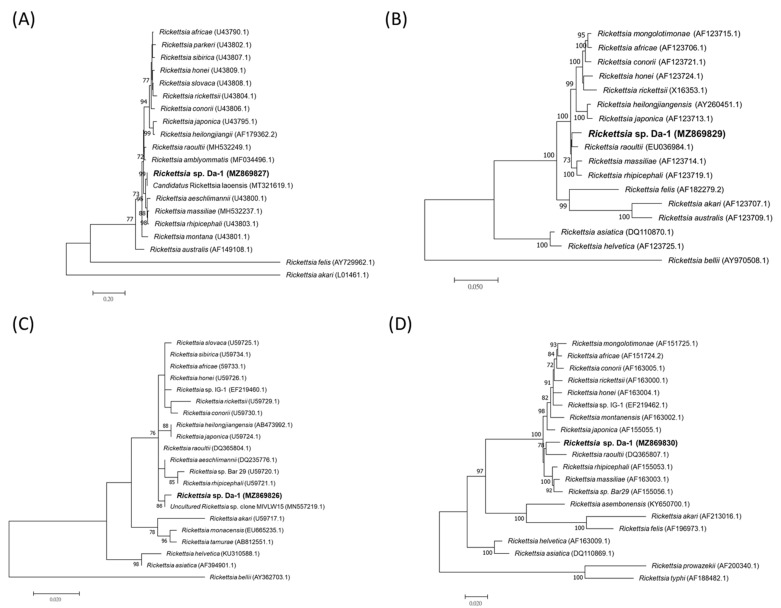
Phylogenetic tree of *Rickettsia* spp. constructed based on (**A**) *ompA* (375 bp); (**B**) *ompB* (2653 bp); (**C**) *gltA* (357 bp); (**D**) *sca4* (2261 bp) sequences. Rickettsial sequences derived from *D. auratus* were compared with those closely related species retrieved from GenBank. The evolutionary relationships were inferred by Maximum Likelihood method with 1000 bootstrap replicates.

*Rickettsia* spp. were detected in 1 (1/1, 100%) *D. auratus*, 7 (7/155, 4.5%) *R. sanguineus*, and 4 (4/24, 16.7%) *R. haemaphysaloides*. The *gltA* amplicons (381 bp) from 6 *R. sanguineus* and 4 *R. haemaphysaloides* were identical to those of *Rickettsia* sp. TwKM01 (AY445819), 99.7% similar to *Rickettsia* spp. from India (MN463671.1, MN463666.1, MN557215.1–MN557217.1, MN557220.1-MN557224.1), and 99.2% similar to *Rickettsia massiliae* (KY640405.1) (Table 4). The *ompA* amplicons (1,073 bp) were identical to those of *Rickettsia* sp. TwKM01 (EF219467.1), 99.5% similar to *Rickettsia rhipicephali* (CP003342.1), and 99.4% similar to *R. massiliae* (U83444.1). The PCR product of *gltA* from 1 *R. sanguineus* was identical to *Rickettsia* sp. TwKM03 (AF540555), *R. felis* (GQ329873.1), and *Rickettsia* sp. RCF01 (GU056201.1). However, the amplified rickettsial fragments from *D. auratus* were relatively close to the sequences of *R. raoultii* and “*Candidatus* Rickettsia laoensis” [36] (Figure 2). Indeed, while the sequences from 5’ end of *ompA* were 100% identical to the “*Candidatus* R. laoensis” isolate (KT753293.1), the sequences from 3’ end of *ompA* were 98.49% similar to *R. raoultii* (AH015609.2). The partial sequences of *ompB* and *sca4* showed similarity of 99.3% and 98.8% to “*Candidatus* R. laoensis” (KT753294.1, KT753292.1), but the reference sequences were rather short (1101/4422 and 820/2472). The *gltA* amplicons were 99.48% similar to *R. raoultii* (MN550897.1) (Table 5). Applying the cut-off values provided by previous publication [37], a divergent strain, *Rickettsia* sp. Da-1, was suggested.

## 3. Discussion

Here we report an extensive study of *Rickettsia* exposure in the northeastern part of Taiwan. The community-based seroepidemiological survey including 1108 residents in Gongliao District revealed seroprevalence of 6.8% (75/1108) for SFG rickettsiae, 4.1% (45/1108) for TG rickettsiae, and 14.0% (115/1108) for *O. tsutsugamushi*. Ticks were collected from dogs living near the seropositive participants to look for potential tick-borne pathogens. *Rickettsia* spp. were detected in 6.5% (12/184) of the ticks. One putative new species similar to *R.* *raoultii* and “*Candidatus* R. laoensis” was discovered in *D. auratus* according to the criteria for molecular identification of *Rickettsia* [37]. Whether these microorganisms lead to human diseases remains to be investigated.

Our study showed 20.0% (222/1108) of the serum samples collected from residents in Gongliao District reacted to at least one group of rickettsiae. Considering IFA of *R. rickettsii* as the gold standard, the sensitivity and specificity of SFG ELISA were 97.4% and 95.8%, respectively. Forty-three of 222 (19.4%) reactive samples were positive to more than one group of rickettsiae. Cross-reactivity has been known to occur between SFG rickettsiae and TG rickettsiae, especially IgG antibodies [38]. Titration of sera was supposed to be performed to reveal differences in antibody titers for the distinction between cross-reactivity [39]. However, the observed IgG titers were generally quite low (≤256), making comparison using titration unrealistic. Therefore, we were unable to determine whether the reactivity was caused by exposure to different groups of rickettsiae or the result of cross-reactions. Moreover, sera from patients with *R. felis* infection were shown to react to *R. rickettsii* and *R. conorii*, and *R. felis* has been proposed to be the major cause of cross-reactions between *R. typhi* and *R. conorii* [40,41]. A study detected *R. felis* infection in 21.6% of patients with rickettsioses in North Taiwan [12]. Hence, *R. felis* exposure was speculated to be responsible for some of the reactions although the current study did not include *R. felis* in the assay. Conversely, cross-reactions between SFG rickettsiae/TG rickettsiae and scrub typhus were less common. Only 5 samples reacted to both *R. rickettsii* and *O. tsutsugamushi*, and 5 samples reacted to both *R. typhi* and *O. tsutsugamushi*. The prevalence of IgG antibodies against SFG rickettsiae, TG rickettsiae, and multiple groups of rickettsiae was significantly associated with older age, implicating progressive exposure to *Rickettsia* spp. Samples collected from Dr. Enjoy’s Clinic tended to have higher seropositive rates probably due to the older average age of the patients. Moreover, a part of the patients from Dr. Enjoy’s Clinic was receiving home-based medical care voluntarily provided by the clinic. These patients generally lived in remote areas and belonged to a disadvantaged minority. As shown in the risk analysis, inhabiting the southwest village, Jilin, was a risk factor for seropositivity.

Although some scenic spots in Gongliao District have become popular attractions for tourists, most parts of the district remained rural. Dogs shuttling back and forth between the fields and human houses, making them a bridge to wildlife as well as their ectoparasitic ticks. In this case, the dogs were considered as sentinels and ticks were collected from the dogs living near seropositive participants. Most collected ticks were the brown dog ticks (*R. sanguineus*) and *R. haemaphysaloides* (155 and 24, respectively), which was generally consistent with other study [42], but species such as *D. auratus*, *H. ornithophila*, and *H. hystricis* also appeared in the collection. *Rickettsia* sp. TwKM01 and *Rickettsia* sp. TwKM03 were detected in *R. sanguineus* and *R. haemaphysaloides*. Phylogenetic analyses revealed *Rickettsia* sp. TwKM01 was most similar to *Rickettsia rhipicephali* [21]. *Rickettsia* sp. TwKM03, a species close to *R. felis*, was first identified in *Leptotrombidium* chigger mites and widely distributed in *I. granulatus* in Hualien, Kinmen, and Matsu in Taiwan [21]. The species has also been found to infect *R. sanguineus* and *Ctenocephalides felis* collected from dogs in Brazil [43]. Moreover, the sequencing results indicated a putative new species, *Rickettsia* sp. Da-1, was identified in *D. auratus*. Pairwise comparison revealed the amplified fragments of *gltA* and *sca4* were 98.4% and 98.8% identical to “*Candidatus* R. laoensis”, respectively, which were lower than the gene sequence-based criteria of identification (99.9% and 99.3%, respectively) despite the 100% similarity of sequences from 5’ end of *ompA* [37]. The amplicons of *ompB* were highly similar to “*Candidatus* R. laoensis”, but the reference sequence was only 1109 base pairs. “*Candidatus* R. laoensis” was first discovered in a pool of *Haemaphysalis* ticks in Laos [36]. Later the *Rickettsia* was observed in 63.6% (14/22 pools) of ticks (*Haemaphysalis bispinosa*, *H. flava*, *H. hystricis*, *Haemaphysalis longicornis*, *Dermacentor atrosignatus*, *D. auratus*, *D. taiwanensis*, *Dermacentor silvarum*) infesting wild boars in Southeast China. However, the study used only partial *ompA* for the screening for infection [44]. The tick host in our study, *D. auratus*, has not previously been documented in Taiwan, but our follow-up studies confirmed the continuous existence of the species on the island (unpulished data). *Dermacentor auratus* has been found to parasitize wild boar, cattle, deer, buffalo, and small mammals in other countries, and cases of human infestation were recorded [45,46]. According to a personal communication, a female residing next to Gongliao District attended a clinic for being attacked by *D. auratus*. Whether *D. auratus* ticks can transmit rickettsiae to humans or small mammals and maintain rickettsiae in nature remains to be studied. On the other hand, one of the limitations of the current study resulted from our method of tick collection. For example, *I. granulatus*, which is abundant and has been shown to carry *Borrelia* and *Rickettsia* [22,47], was left out along with many other ticks known to occur in Taiwan. However, *I. granulatus* mainly parasitizes small mammals and there is no record of it attacking humans in the Australasian Zoogeographic Region [48]. Considering the terrain, the animal contact history, and the less active lifestyle of the participants, ticks were collected only from dogs in the study. Further surveys of ticks using different collection methods would provide more information regarding ticks and tick-borne pathogens in the region.

Tick-borne rickettsioses have been considered as important infectious diseases in the Western World. For example, Rocky Mountain spotted fever and human granulocytic anaplasmosis are nationally notifiable diseases in the USA [49]. Thirteen EU countries have established a surveillance system at the national level and reporting of rickettsioses are mandatory [50]. In Asia, Japanese spotted fever is a notifiable infectious disease in Japan, with approximately 200–300 cases reported annually and once a seropositive rate of 45.1% in Okinawa [51]. New pathogenic species, such as *Rickettsia heilongjiangensis*, *R. helvetica*, and *Rickettsia tamurae*, and other species with unknown pathogenicity, such as *Rickettsia asiatica* and “*Candidatus* Rickettsia tarasevichiae”, have been identified after *R. japonica* [52,53,54,55,56]. In South Korea, Thailand, Malaysia, and Laos, seroprevalence for SFG rickettsiae has been described as 16.2–19.9%, 0.8–4%, 42.5%, and 2.6% in patients with acute febrile illness, respectively [57,58,59,60,61,62]. The seroprevalence was reported as 1.7% and 10.4–20.4% in healthy populations in Vietnam and Indonesia, respectively [63,64]. Spotted fever is a common disease in China, and the seroprevalence has been demonstrated as high as 54.8% in the healthy population in Eastern China [65]. New species, including *R. heilongjiangensis* and *Rickettsia sibirica mongolotimonae*, have also been identified [37,66].

In Taiwan, tick-borne SFG rickettsioses are still poorly understood. Our findings confirmed the exposure of SFG rickettsiae, with a seropositive rate of 6.8% in residents of the northeastern part of the island. *Rickettsia* spp. and other endosymbionts were identified in ticks, implying the existence of transmission cycles. Although the pathogenicity of these rickettsiae remained uncertain, SFG rickettsiae infection should be considered in the differential diagnosis of fever of unknown origin in addition to scrub typhus. This work emphasized the risk of tick-borne rickettsioses. Residents as well as tourists should wear personal protection equipment or repellent when engaging in outdoor activities, and practicing tick control in dogs is advised. Further investigations will focus on the patients to detect and isolate SFG rickettsiae for the verification of etiological agents of febrile illnesses. Other tick-borne pathogens, such as severe fever with thrombocytopenia syndrome virus, will also be explored.

## 4. Materials and Methods

### 4.1. Study Setting and Human Subject

Gongliao District is a rural district located in the northeastern part of Taiwan. The district comprises an area of approximately 99.97 square kilometers which is divided into 11 villages. The population was 13,970 in 2008 with 73.6% of the inhitants dwelling in Zhenli, Renli, Hemei, Fulong, Goungliao, and Fulian villages. The terrain is generally hilly. Facing the Pacific Ocean, Gongliao District is famous for its golden-sand beach, the Fulong beach, and other scenic settings as well as old hiking trails. Tourists come to the district for seafood or recreation activities, such as surfing, windsurfing, fishing, hiking, and the music festival.

Human subjects were recruited through 3 sources, including healthy individuals attending physical examinations in a group practice center, non-febrile patients visiting the group practice center, and patients visiting or receiving home-based medical care provided by a clinic. Residents of Gongliao District, which covered workers who had been staying there for over 3 months, were invited to particpate in the study. Blood samples were collected via venipuncture after obtaining the subjects’ consent and left to stand for 30 min at room temperature. Sera were separated by centrifugation, aliquoted, and carried back to the laboratory on ice. The samples were then kept frozen at −20°C until further analyses. Meanwhile, willing participants were asked to fill out paper-based questionnaires to provide demographic information for further risk analysis. All participants did not exhibit typical signs of acute infections at the time of sampling.

### 4.2. Serology

#### 4.2.1. Screening of Sera by ELISA for SFG Rickettsiae Exposure

Sera were tested for the presence of IgG antibodies agaist SFG rickettsiae using Panbio Spotted Fever Group IgG ELISA (Panbio, Brisbane, Australia). The assay was performed with serum samples diluted in serum diluent to 1:100. The PANBIO units were determined by dividing the sample absorbance by the average absorbance of the cut-off calibrator providede and multiplying by 10. A PANBIO unit >11 indicated a positive result, suggesting presence of detectable IgG to SFG rickettsiae.

#### 4.2.2. Detection of IgG against SFG Rickettsiae, TG Rickettsiae, and *O. tsutsugamushi* by IFA

IgG antibodies against SFG rickettsiae and TG rickettsiae in the serum samples were examined by a commercially available IFA kit containg antigens of *R. rickettsii* and *R. typhi* (IF0100G, Focus Technologies Inc, Cypress, CA, USA) according to the manufacturer’s instructions. Antibodies against tick-borne SFG rickettsiae were further screened using *R. conorii* Substrate Slide (IF0104, Focus Technologies Inc, Cypress, CA, USA) [19]. Briefly, sera were diluted to 1:32 and applied to the wells. After 30 min of incubation at 37°C, the slides were washed with PBS. Fluorescein isothiocyanate (FITC) conjugated goat anti-human IgG/A/M (ThermoFisher Scientific Inc., Camarillo, CA, USA) was then added. After incubation, the slides were washed, dried, and mounted. The results were read at a magnification of 400× with a fluorescence microscope (Leica Microsystem, Singapore) by two technicians independently. An antibody titer of ≥1:64 was considered as a positive reaction.

Antibodies against *O. tsutsugamushi* were screened by indirect IFA using slides coated with whole-cell antigens of the Karp strain as described [67,68]. A titer ≥ 1:64 was considered seropositive and indicated potential exposure to *O. tsutsugamushi*.

### 4.3. Collection of Ticks and Tick Species Identification

Ticks parasitizing dogs were collected within a 2 km radius from the houses of participants who tested positive for SFG rickettsiae antibodies during August to December, 2009. Ticks were removed by forceps or a tick twister after obtaining agreement from the dog owners and stored in 70% ethanol. Species identification was carried out by oberserving the taxonomic characteristics under a dissecting microscope [69,70,71,72]. Molecular identification targeting the mitochondrial 16S rDNA and *COI* was applied to nymphs whose morphological features were not fully developed and adults whose key characteristics were undistinguishable due to damages during removal or engorgement with blood for confimative purpose [69,73,74]. However, the coding sequences of *COI* were not efficiently amplified in nearly half of the specimens by PCR. Therefore, molecular identification was primarily dependent on the amplicons of the 16S rDNA. In addition, the 16S rDNA of ticks other than *R. sanguineus* and *R. haemaphysaloides* were sequenced to provide further information for the genetic charateraterization.

### 4.4. Detection of Potential Tick-Borne Pathogens

The collected ticks were cut symmetrically into halves, and a half of each tick was used in DNA extraction with Genomic DNA Mini Kit (Geneaid, Taipei, Taiwan). PCR was performed to detect potential pathogens carried by ticks. Identification of rickettsial infections was achieved by primers targeting *ompA*, *ompB*, *gltA*, and *sca4* as previously described [75,76,77,78,79]. PCR products were sent to Mission Biotech (Taipei, Taiwan) for Sanger sequencing in both forward and reverse directions.

### 4.5. Phylogenetic Analysis

Sequences of the amplicons were first examined with Seqman 7.1.0 (Lasergene, Madison, WI, USA). Then the sequences were seperately aligned against those closely related species found in GenBank using the Clustal W application within BioEdit 7.2.0. Phylogenetic trees were constructed based on the alignment using a maximum likelihood inference with 1,000 bootstrap replicates in MEGA7 [80]. Novelty of *Rickettsia* sp. was determined by the sequence identities of the pairwise comparison of *gltA*, *ompA*, *ompB*, and *sca4* sequences with their closest related species [37].

### 4.6. Statistical Analysis

The demographic information and serological results of participants were inputted to a Microsofft Excel (Microsoft Corporation, Washington, DC, USA) datasheet for further processing. A Kruskal-Wallis test (non-parametric test) was done to compare the geometric ratios between groups with a significance level of 0.05. Associations between subject characteristics and serological results were studied using univariable and multivariable logistic regression, using the odds ratios and 95% Confidence Interval (CI) as measurement. All variables were treated as categorical. Multivariable analysis included variables which were significantly associated with seropositivity in the univariable analysis. Effect modification and interaction were assessed for each covariate associated with seropositivity. Statistical analyses were performed using SPSS version 20.0 software (SPSS Inc., Chicago, IL, USA).

## Figures and Tables

**Table 1:** Characteristics of study population.

Variables	Annual Health Exam (n = 260)	Patients Visiting the Group Practice Center (n = 557)	Patients Visiting Dr. Enjoy’s Clinic (n = 291)	*p* Value
Gender				<0.01
Male	104	327	106	
Female	156	230	185	
**Age (mean ± SD)**	55.1 ± 15.2	58.2 ± 20.4	63.3 ± 16.1	<0.01
**Village**				<0.01
Gongliao (GL)	12	24	106	
Jilin (JL)	1	9	24	
Shuangyu (SY)	10	29	41	
Longgang (LG)	0	4	46	
Longmen (LM)	8	25	14	
Fulong (FL)	23	65	21	
Renli (RL)	83	137	18	
Zhenli (ZL)	78	149	11	
Fulian (FN)	14	31	6	
Meifeng (MF)	29	38	3	
Hemei (HM)	2	46	1	

**Table 2:** Residents in Gongliao District with antibodies against spotted fever group rickettisae, typhus group rickettsiae, and scrub typhus examined by SFGR ELISA and IFA.

	**SFGR ELISA** **(n = 118)**	IFA				
		*R. rickettsia* (n = 77)	*R. conorii* (n = 68)	*R. typhi* (n = 45)	*O. tsutsugamushi* (n = 155)	*R. rickettsii + R. typhi* (n = 23)
SFGR ELISA	-	75	68	26	15	23
IFA						
*R. rickettsii*	75	-	62	23	5	-
*R. conorii*	68	62	-	24	9	23
*R. typhi*	26	23	24	-	5	-
*O. tsutsugamushi*	15	5	9	5	-	10

**Table 3:** Seroprevalence and logistic regression analysis of associated possible correlates for SFG rickettsiae exposure in subjects in Gongliao District, New Taipei City, Taiwan.

Variables	No. of Samples Tested	No. (%) of Positive Samples	Univariate Regression Analysis	Multiple Regression Analysis
			OR (95% CI)	OR (95% CI)
**Gender**				
Male	537	39 (7.3)	Reference	ND
Female	571	36 (6.3)	0.9 (0.5–1.4)	ND
**Age**				
<65 yr	564	21 (3.7)	Reference	Reference
*≥*65 yr	544	54 (9.9)	2.9 (1.7–4.8) ^***^	2.1 (1.2–3.8) ^*^
**Sampling site**				
Annual health exam	260	10 (3.8)	Reference	Reference
Group practice center	557	41 (7.4)	2.4 (1.1–5.2) ^*^	1.7 (0.7–3.9)
Dr. Enjoy’s Clinic	291	24 (8.2)	3.4 (1.5–7.5) ^**^	2.2 (0.8–5.8)
**Village**				
Gongliao (GL)	142	9 (6.3)	Reference	Reference
Jilin (JL)	34	7 (20.6)	3.8 (1.3–11.2) ^*^	3.3 (1.1–10.1) ^*^
Shuangyu (SY)	80	8 (10.0)	1.6 (0.6–4.4)	1.5 (0.5–4.1)
Longgang (LG)	50	5 (10.0)	1.6 (0.5–5.2)	1.4 (0.4–4.5)
Longmen (LM)	47	4 (8.5)	1.4 (0.4–4.7)	1.6 (0.4–5.7)
Fulong (FL)	109	7 (6.4)	1.0 (0.4–2.8)	1.2 (0.4–3.7)
Renli (RL)	238	11 (4.6)	0.7 (0.3–1.8)	0.9 (0.3–2.7)
Zhenli (ZL)	238	10 (4.2)	0.6 (0.3–1.6)	0.8 (0.3–2.4)
Fulian (FN)	51	4 (7.8)	1.3 (0.4–4.3)	1.9 (0.5–7.3)
Meifeng (MF)	70	6 (8.6)	1.4 (0.5–4.1)	1.6 (0.5–5.3)
Hemei (HM)	49	4 (8.2)	1.3 (0.4–4.5)	1.8 (0.5–7.2)
**Occupation**				
NA	239	22 (9.2)	Reference	Reference
Agricultural worker	67	6 (9.0)	1.0 (0.4–2.5)	0.7 (0.2–1.9)
Housemaker	331	29 (8.8)	0.9 (0.5–1.7)	0.9 (0.4–1.8)
Industrial laborer	191	7 (3.7)	0.4 (0.2–0.9) ^*^	0.6 (0.2–1.5)
Businessman	57	0 (0.0)	0.0 (0.0)	0.0 (0.0)
Government official	27	1 (3.7)	0.4 (0.0–2.9)	0.5 (0.1–4.4)
Teacher	7	1 (14.3)	1.6 (0.2–14.3)	2.7 (0.3–25.9)
Armed force occupation	1	0 (0.0)	0.0 (0.0)	0.0 (0.0)
Student	15	1 (6.7)	0.7 (0.1–5.6)	0.8 (0.1–7.2)
Other	173	8 (4.6)	0.5 (0.2–1.2)	0.6 (0.2–1.4)

^*^p < 0.05; ^**^ *p* < 0.01; ^***^ *p* < 0.001; OR: odds ratio; CI: confidence interval; NA: not available; ND: not done.

**Table 4:** Detection of *Rickettsia* spp. in ticks parasitizing dogs in Gongliao District, New Taipei City, Taiwan.

Tick Species (Accession No.)	No. Ticks (Female, Male, Nymph)	*Rickettsia* spp.	
		Positive Rate %(Positive/Tested)	Accession No.
*Dermacentor auratus* (MZ823781)	1 (1, 0, 0)	100.0 (1/1)	MZ869826 MZ869827 MZ869828 MZ869829 MZ869830
*Haemaphysalis hystricis* (MZ823778)	3 (2, 1, 0)	0.0 (0/3)	
*Haemaphysalis ornithophila* (MZ823776)	1 (0, 1, 0)	0.0 (0/1)	
*Rhipicephalus sanguineus*	155 (52, 37, 66)	4.5 (7/155)	AY445819 ^1^ AF540555 ^1^ EF219467.1 ^1^
*Rhipicephalus haemaphysaloides*	24 (4, 7, 13)	16.7 (4/24)	AY445819 ^1^
Total	184	6.5 (12/184)	

^1^ Detected sequences were identical to previously published ones [21].

**Table 5:** Pairwise comparison of partial sequences from *gltA*, *ompA*, *ompB*, and *sca4* between amplicons from *D. auratus* and references in GenBank. Cut-off values for molecular species identification was included.

Gene	% Pairwise Nucleotide Sequence Identity to Closest Neighbors (Accession No.)	No. Matching Nucleotides/Total	Cutoff Values [37]
*gltA*	99.74% to Uncultured *Rickettsia* sp. clone MIVLW15/2017 (MN557219.1) 99.74% to Uncultured bacterium clone HHMJ7 (KC566999.1) 99.48% to *R. raoultii* isolate N42 (MN550897.1) 98.41% to “*Candidatus* R. laoensis” (KT753290.1)	383/384 381/382 382/384 124/126	99.9%
5ʹ end of *ompA*	100.0% to “*Candidatus* R. laoensis” isolate MHS2019/12 (MT321619) 98.55% to “*Candidatus* R. laoensis” isolate MIVLW15/2017 (MK905251.1) 97.64% to *R. raoultii* isolate z164 (MH532249.1)	551/551 543/551 538/551	98.8%
3ʹ end of *ompA*	98.49% to *Rickettssia* sp. RpA4 (AH009131.2) 98.49% to *R. raoultii* strain Marne (AH015609.2) 98.40% to *R. raoultii* isolate Tomsk (MK304548.1)	3134/3182 3134/3182 3132/3183	
*ompB*	97.99% to *R. raoultii* strain Khabarovsk (CP010969.1) 97.96% to *R. raoultii* strain IM16 (CP019435.1) 97.94% to *R. raoultii* strain Khabarovsk (DQ365798.1) 99.28% to “*Candidatus* R. laoensis” (KT753294.1)	4333/4422 4332/4422 4334/4425 1101/1109	99.2%
*sca4*	98.01% to *R. montanensis* str. OSU 85-930 (CP003340.1) 97.82% to *R. raoultii* isolate Tomsk (MK304550.1) 97.73% to *R. raoultii* isolate Nsk862 (MT253668.1) 98.80% to “*Candidatus* R. laoensis” (KT753292.1)	2417/2466 2418/2472 2416/2472 820/830	99.3%

## Data Availability

The DNA sequences generated during this study are openly available in GenBank. Other primary data are available on request from the corresponding author. Certain data are not publicly available due to ethical concern.

## References

[1] Walker D., Baron S. (1996). Rickettsiae. Medical Microbiology.

[2] Brezina R., Murray E., Tarizzo M., Bögel K. (1973). Rickettsiae and rickettsial diseases. Bull. World Health Organ..

[3] Tamura A., Ohashi N., Urakami H., Miyamura S. (1995). Classification of *Rickettsia tsutsugamushi* in a new genus, *Orientia* gen. nov., as *Orientia tsutsugamushi* comb. nov. Int. J. Syst. Bacteriol..

[4] Gillespie J.B., Beier M.S., Rahman M., Ammerman N., Shallom J., Purkayastha A., Sobral B., Azad A. (2007). Plasmids and rickettsial evolution: Insight from *Rickettsia felis*. PLoS ONE.

[5] Sekeyová Z., Danchenko M., Filipčík P., Fournier P. (2019). Rickettsial infections of the central nervous system. PLoS Negl. Trop. Dis..

[6] Taiwan Centers for Disease Control. Taiwan National Infectious Disease Statistics System. https://store.neurosky.com/pages/mindwave.

[7] Tsai K., Chung L., Chien C., Tung Y., Wei H., Yen T., Shu P., Wang H. (2019). Human granulocytic anaplasmosis in Kinmen, an offshore island of Taiwan. PLoS Negl. Trop. Dis..

[8] Yen T., Tung Y., Wang H., Tsai K. (2020). Detection of *Ehrlichia chaffeensis* in a febrile patient in Kinmen, an offshore island of Taiwan. J. Formos Med. Assoc..

[9] Peng S., Yang S., Ho Y., Chen H., Shu P. (2019). Human case of *Ehrlichia chaffeensis* infection, Taiwan. Emerg. Infect. Dis..

[10] Tsai K., Lu H., Tsai J., Yu S., Huang J., Shu P. (2008). Human case of *Rickettsia felis* infection, Taiwan. Emerg. Infect. Dis..

[11] Lai C., Chang L., Lin J., Tsai K., Hung Y., Kuo L., Lin H., Chen Y. (2014). Human spotted fever group rickettsioses are underappreciated in southern Taiwan, particularly for the species closely-related to *Rickettsia felis*. PLoS ONE.

[12] Yang W., Hsu M., Shu P., Tsai K., Fang C. (2021). Neglected human *Rickettsia felis* infection in Taiwan: A retrospective seroepidemiological survey of patients with suspected rickettsioses. PLoS Negl. Trop. Dis..

[13] Tsai K., Lu H., Huang J., Fournier P., Mediannikov O., Raoult D., Shu P. (2009). African tick bite fever in a Taiwanese traveler returning from South Africa: Molecular and serologic studies. Am. J. Trop. Med. Hyg..

[14] Kuo C., Shu P., Mu J., Lee P., Wu Y., Chung C., Wang H. (2015). Widespread *Rickettsia* spp. Infections in Ticks (Acari: Ixodoidea) in Taiwan. J. Med. Entomol..

[15] Kuo C., Huang J., Lin T., Wang H. (2012). Detection of *Rickettsia* spp. and host and habitat associations of fleas (Siphonaptera) in eastern Taiwan. Med. Vet. Entomol..

[16] Kuo C., Lin Y., Yao C., Shih H., Chung L., Liao H., Hsu Y., Wang H. (2017). Tick-borne pathogens in ticks collected from birds in Taiwan. Parasit Vectors.

[17] Shih C., Yang P., Chao L. (2021). Molecular Detection and genetic identification of *Rickettsia* infection in *Ixodes granulatus* ticks, an incriminated vector for geographical transmission in Taiwan. Microorganisms.

[18] Kuo C., Shu P., Mu J., Wang H. (2015). High prevalence of *Rickettsia* spp. infections in small mammals in Taiwan. Vector Borne Zoonotic Dis..

[19] Kuo C., Huang C., Wang H. (2011). Identification of potential hosts and vectors of scrub typhus and tick-borne spotted fever group rickettsiae in eastern Taiwan. Med. Vet. Entomol..

[20] Hsu Y., Lin C., Chome l.B., Tsai K., Wu W., Huang C., Chang C. (2011). Identification of *Rickettsia felis* in fleas but not ticks on stray cats and dogs and the evidence of *Rickettsia rhipicephali* only in adult stage of *Rhipicephalus sanguineus* and *Rhipicephalus haemaphysaloides*. Comp. Immunol. Microbiol. Infect. Dis..

[21] Tsui P., Tsai K., Weng M., Hung Y., Liu Y., Hu K., Lien J., Lin P., Shaio M., Wang H. (2007). Molecular detection and characterization of spotted fever group rickettsiae in Taiwan. Am. J. Trop. Med. Hyg..

[22] Tsai K., Wang H., Chen C., Huang J., Lu H., Su C., Shu P. (2008). Isolation and identification of a novel spotted fever group rickettsia, strain IG-1, from *Ixodes granulatus* ticks collected on Orchid Island (Lanyu), Taiwan. Am. J. Trop. Med. Hyg..

[23] Rochlin I., Toledo A. (2020). Emerging tick-borne pathogens of public health importance: A mini-review. J. Med. Microbiol..

[24] Guglielmone A., Robbins R., Apanaskevich D., Petney T., Estrasa-Pena A., Horak I., Shao R., Barker S. (2010). The Argasidae, Ixodidae and Nuttalliellidae (Acari: Ixodida) of the world: A list of valid species names. Zootaxa.

[25] Robbins R. (2005). The ticks (Acari: Ixodida: Argasidae, Ixodidae)of Taiwan: A synonymic checklist. Proc. Entomol. Soc. Wash..

[26] Tsai Y., Shyu C., Yao C., Lin J. (2012). The ixodid ticks collected from dogs and other animals in Taiwan and Kinmen Island. Int. J. Acarol..

[27] Kwak M., Kuo C., Chu H. (2020). First record of the sea snake tick *Amblyomma nitidum* Hirst and Hirst, 1910 (Acari: Ixodidae) from Taiwan. Ticks Tick Borne Dis..

[28] Kuo C., Huang J., Chien C., Shih H., Wang H. (2018). First molecular detection of *Anaplasma phagocytophilum* in the hard tick *Rhipicephalus haemaphysaloides* in Taiwan. Exp. Appl. Acarol..

[29] Chao L., Shih C. (2016). Molecular analysis of *Rhipicephalus sanguineus* (Acari: Ixodidae), an incriminated vector tick for Babesia vogeli in Taiwan. Exp. Appl. Acarol..

[30] Chao L., Liu L., Ho T., Shih C. (2014). First detection and molecular identification of *Borrelia garinii* spirochete from *Ixodes ovatus* tick ectoparasitized on stray cat in Taiwan. PLoS ONE.

[31] Shih C., Wang J., Chao L., Wu T. (1998). Lyme disease in Taiwan: First human patient with characteristic erythema chronicum migrans skin lesion. J. Clin. Microbiol..

[32] Shih C., Liu L., Chung W., Ong S., Wang C. (1997). Human babesiosis in Taiwan: Asymptomatic infection with a *Babesia microti*-like organism in a Taiwanese woman. J. Clin. Microbiol..

[33] Lin T., Ou S., Maeda K., Shimoda H., Chan J., Tu W., Hsu W., Chou C. (2020). The first discovery of severe fever with thrombocytopenia syndrome virus in Taiwan. Emerg. Microbes Infect..

[34] Peng S., Yang S., Tang S., Wang T., Hsu T., Su C., Chen M., Shimojima M., Yoshikawa T., Shu P. (2020). Human case of severe fever with thrombocytopenia syndrome virus infection, Taiwan, 2019. Emerg. Infect. Dis..

[35] Takada N., Fujita H., Yano Y., Huang W., Khamboonruang C. (1993). Serosurveys of spotted fever and murine typhus in local residents of Taiwan and Thailand compared with Japan. Southeast. Asian J. Trop. Med. Public Health.

[36] Taylor A., Vongphayloth K., Vongsouvath M., Grandadam M., Brey P., Newton P., Sutherland I., Dittrich S. (2016). Large-scale survey for tickborne bacteria, Khammouan Province, Laos. Emerg. Infect. Dis..

[37] Fournier P., Dumler J., Greub G., Zhang J., Wu Y., Raoult D. (2003). Gene sequence-based criteria for identification of new *Rickettsia* isolates and description of *Rickettsia heilongjiangensis* sp. nov. J. Clin. Microbiol..

[38] Ormsbee R., Peacock M., Philip R., Casper E., Plorde J., Gabre-Kidan T., Wright L. (1978). Antigenic relationships between the typhus and spotted fever groups of rickettsiae. Am. J. Epidemiol..

[39] Pérez-Arellano J., Fenollar F., Angel-Moreno A., Bolaños M., Hernández M., Santana E., Hemmersbach-Miller M., Martín A., Raoult D. (2005). Human *Rickettsia felis* infection, Canary Islands, Spain. Emerg. Infect. Dis..

[40] Raoult D., La Scola B., Enea M., Fournier P., Roux V., Fenollar F., Galvao M., de Lamballerie X. (2001). A flea-associated *Rickettsia* pathogenic for humans. Emerg. Infect. Dis..

[41] Znazen A., Rolain J., Hammami A., Jemaa M., Raoult D. (2006). *Rickettsia felis* infection, Tunisia. Emerg. Infect. Dis..

[42] Chao L., Hsieh C., Ho T., Shih C. (2019). First zootiological survey of hard ticks (Acari: Ixodidae) infesting dogs in northern Taiwan. Exp. Appl. Acarol..

[43] Gehrke F., Gazeta G., Souza E., Ribeiro A., Marrelli M., Schumaker T. (2009). *Rickettsia rickettsii*, *Rickettsia felis* and *Rickettsia* sp. TwKM03 infecting *Rhipicephalus sanguineus* and *Ctenocephalides felis* collected from dogs in a Brazilian spotted fever focus in the State of Rio De Janeiro/Brazil. Clin. Microbiol. Infect..

[44] Wang X., Sun X., Sun Y., Chen K., Zhang K., Xu W., Fan K., Lin W., Chen T., Lin X. (2020). Identification and molecular analysis of *Ixodid* ticks (Acari: Ixodidae) infesting wild boars (*Sus scrofa*) and tick-borne pathogens at the Meihua mountain of southwestern Fujian, China. Vet. Parasitol. Reg. Stud. Rep..

[45] Ajithkumar K., Ravindran R., Ghosh S. (2012). *Dermacentor auratus* Supino, 1897 (Acarina, Ixodidae) reported from Wayanad, Kerala. Indian J. Med. Res..

[46] Kwak M., Chavatte J., Chew K., Lee B. (2021). Emergence of the zoonotic tick *Dermacentor* (*Indocentor*) *auratus* Supino, 1897 (Acari: Ixodidae) in Singapore. Ticks Tick Borne Dis.

[47] Chao L., Wu W., Shih C. (2009). Molecular analysis of *Ixodes granulatus*, a possible vector tick for *Borrelia burgdorferi* sensu lato in Taiwan. Exp. Appl Acarol.

[48] Guglielmone A., Robbins R. (2018). Hard ticks (Acari: Ixodida: Ixodidae) parasitizing humans: A global overview.

[49] McNabb S., Jajosky R., Hall-Baker P., Adams D., Sharp P., Worshams C., Anderson W., Javier A., Jones G., Nitschke D. (2008). Summary of notifiable diseases—United States, 2006. MMWR Morb Mortal Wkly. Rep..

[50] ECDC (2013). Epidemiological Situation of Rickettsioses in EU/EFTA Countries.

[51] Satoh H., Tsuneki A., Inokuma H., Kumazawa N., Jahana Y., Kiyuuna T., Okabayashi T., Muramatsu Y., Ueno H., Morita C. (2001). Seroprevalence of antibodies against spotted fever group rickettsia among dogs and humans in Okinawa, Japan. Microbiol. Immunol..

[52] Ando S., Kurosawa M., Sakata A., Fujita H., Sakai K., Sekine M., Katsumi M., Saitou W., Yano Y., Takada N. (2010). Human *Rickettsia heilongjiangensis* infection, Japan. Emerg. Infect. Dis..

[53] Imaoka K., Kaneko S., Tabara K., Kusatake K., Morita E. (2011). The first human case of *Rickettsia tamurae* infection in Japan. Case Rep. Dermatol..

[54] Fujita H., Fournier P., Takada N., Saito T., Raoult D. (2006). *Rickettsia asiatica* sp. nov., isolated in Japan. Int. J. Syst. Evol. Microbiol..

[55] Inokuma H., Ohashi M., Tanabe S., Miyahara K., Jilintai (2007). Prevalence of tick-borne *Rickettsia* and *Ehrlichia* in *Ixodes persulcatus* and *Ixodes ovatus* in Tokachi district, Eastern Hokkaido, Japan. J. Vet. Med. Sci..

[56] Fournier P., Takada N., Fujita H., Raoult D. (2006). *Rickettsia tamurae* sp. nov., isolated from *Amblyomma testudinarium* ticks. Int. J. Syst. Evol. Microbiol..

[57] Jang W., Kim J., Choi Y., Jung K., Kim Y., Lee S., Choi M., Kim I., Walker D., Park K. (2004). First serologic evidence of human spotted fever group rickettsiosis in Korea. J. Clin. Microbiol..

[58] Jang W., Choi Y., Kim J., Jung K., Ryu J., Lee S., Yoo C., Paik H., Choi M., Park K. (2005). Seroepidemiology of spotted fever group and typhus group rickettsioses in humans. Microbiol. Immunol..

[59] Strickman D., Tanskul P., Eamsila C., Kelly D. (1994). Prevalence of antibodies to rickettsiae in the human population of suburban Bangkok. Am. J. Trop. Med. Hyg..

[60] Bhengsri S., Baggett H., Edouard S., Dowell S., Dasch G., Fisk T., Raoult D., Parola P. (2016). Sennetsu neorickettsiosis, spotted fever group, and typhus group rickettsioses in three provinces in Thailand. Am. J. Trop. Med. Hyg..

[61] Tay S., Ho T., Rohani M., Devi S. (2000). Antibodies to *Orientia tsutsugamushi*, *Rickettsia typhi* and spotted fever group rickettsiae among febrile patients in rural areas of Malaysia. Trans. R Soc. Trop. Med. Hyg..

[62] Phongmany S., Rolain J., Phetsouvanh R., Blacksell S., Soukkhaseum V., Rasachack B., Phiasakha K., Soukkhaseum S., Frichithavong K., Chu V. (2006). Rickettsial infections and fever, Vientiane, Laos. Emerg. Infect. Dis..

[63] Trung N., Hoi L., Thuong N., Toan T., Huong T., Hoa T., Fox A., Kinh N., van Doorn H., Wertheim H. (2017). Seroprevalence of scrub typhus, typhus, and spotted fever among rural and urban populations of Northern Vietnam. Am. J. Trop. Med. Hyg..

[64] Richards A., Ratiwayanto S., Rahardjo E., Kelly D., Dasch G., Fryauff D., Bangs M. (2003). Serologic evidence of infection with ehrlichiae and spotted fever group rickettsiae among residents of Gag Island, Indonesia. Am. J. Trop. Med. Hyg..

[65] Li J., Hu W., Wu T., Li H., Hu W., Sun Y., Chen Z., Shi Y., Zong J., Latif A. (2018). Japanese Spotted Fever in Eastern China, 2013. Emerg. Infect. Dis..

[66] Fournier P., Gouriet F., Brouqui P., Lucht F., Raoult D. (2005). Lymphangitis-associated rickettsiosis, a new rickettsiosis caused by *Rickettsia sibirica mongolotimonae*: Seven new cases and review of the literature. Clin. Infect. Di.s.

[67] Yen T., Zhang Z., Chao C., Ching W., Shu P., Tseng L., Carvalho A., Tsai K. (2019). Serologic evidence for *Orientia* exposure in the Democratic Republic of Sao Tome and Principe. Vector Borne Zoonotic Dis..

[68] Demma L., McQuiston J., Nicholson W., Murphy S., Marumoto P., Sengebau-Kingzio M., Kuartei S., Durand A., Swerdlow D. (2006). Scrub typhus, Republic of Palau. Emerg. Infect. Dis..

[69] Walker A., Walker A. (1994). Ticks-Ixodida. The Arthropods of Humans and Domestic Animals.

[70] Walker J., Keirans J., Horak I. (2000). The Genus Rhipicephalus (Acari, Ixodidae): A Guide to the Brown Ticks of the World.

[71] Yamaguti N., Tipton V., Keegan H., Toshiaoka S. (1971). Ticks of Japan, Korea, and the Ryukyu islands. Brigh Young Univ. Sci. Bull. Biol. Ser..

[72] Teng K., Jiang Z. (1991). Acari: Ixodidae.

[73] Black W.t., Piesman J. (1994). Phylogeny of hard- and soft-tick taxa (Acari: Ixodida) based on mitochondrial 16S rDNA sequences. Proc. Natl. Acad. Sci. USA.

[74] Folmer O., Black M., Hoeh W., Lutz R., Vrijenhoek R. (1994). DNA primers for amplification of mitochondrial cytochrome c oxidase subunit I from diverse metazoan invertebrates. Mol. Mar. Biol. Biotechnol..

[75] Fournier P., Roux V., Raoult D. (1998). Phylogenetic analysis of spotted fever group rickettsiae by study of the outer surface protein rOmpA. Int. J. Syst. Bacteriol..

[76] Roux V., Raoult D. (2000). Phylogenetic analysis of members of the genus *Rickettsia* using the gene encoding the outer-membrane protein rOmpB (ompB). Int. J. Syst. Evol. Microbiol..

[77] Roux V., Rydkina E., Eremeeva M., Raoult D. (1997). Citrate synthase gene comparison, a new tool for phylogenetic analysis, and its application for the rickettsiae. Int. J. Syst. Bacteriol..

[78] Sekeyova Z., Roux V., Raoult D. (2001). Phylogeny of *Rickettsia* spp. inferred by comparing sequences of ‘gene D’, which encodes an intracytoplasmic protein. Int. J. Syst. Evol. Microbiol..

[79] Hsi T., Hsiao S., Minahan N., Yen T., de Assunção Carvalho A., Raoult D., Fournier P., Tsai K. (2020). Seroepidemiological and molecular investigation of spotted fever group rickettsiae and *Coxiella burnetii* in Sao Tome Island: A One Health approach. Transbound Emerg. Dis..

[80] Kumar S., Stecher G., Tamura K. (2016). MEGA7: Molecular evolutionary genetics analysis version 7.0 for bigger datasets. Mol. Biol. Evol..

